# N-Phenylpyridine-3-Carboxamide and 6-Acetyl-1H-Indazole Inhibit the RNA Replication Step of the Dengue Virus Life Cycle

**DOI:** 10.1128/aac.01331-22

**Published:** 2023-01-26

**Authors:** Aïssatou Aïcha Sow, Felix Pahmeier, Yann Ayotte, Anaïs Anton, Clément Mazeaud, Tania Charpentier, Léna Angelo, Simon Woo, Berati Cerikan, Darryl Falzarano, Levon Abrahamyan, Alain Lamarre, Patrick Labonté, Mirko Cortese, Ralf Bartenschlager, Steven R. LaPlante, Laurent Chatel-Chaix

**Affiliations:** a Centre Armand-Frappier Santé Biotechnologie, Institut National de la Recherche Scientifique, Laval, Quebec, Canada; b Department of Infectious Diseases, Molecular Virology, Center for Integrative Infectious Disease Research (CIID), Heidelberg University, Heidelberg, Germany; c Vaccine and Infectious Disease Organization (VIDO), University of Saskatchewan, Saskatoon, Saskatchewan, Canada; d Department of Veterinary Microbiology, University of Saskatchewan, Saskatoon, Saskatchewan, Canada; e Faculty of Veterinary Medicine, University of Montreal, Saint-Hyacinthe, Quebec, Canada; f Telethon Institute of Genetics and Medicine, Naples, Italy; g German Center for Infection Research (DZIF), Heidelberg partner site, Heidelberg, Germany; h Center of Excellence in Orphan Diseases Research-Foundation Courtois, Quebec, Canada; i Réseau Intersectoriel de Recherche en Santé de l’Université du Québec, Quebec, Canada

**Keywords:** antivirals, dengue virus, West Nile virus, Zika virus, SARS-CoV-2

## Abstract

Dengue virus (DENV) is a *Flavivirus* that causes the most prevalent arthropod-borne viral disease. Clinical manifestation of DENV infection ranges from asymptomatic to severe symptoms that can lead to death. Unfortunately, no antiviral treatments against DENV are currently available. In order to identify novel DENV inhibitors, we screened a library of 1,604 chemically diversified fragment-based compounds using DENV reporter viruses that allowed quantification of viral replication in infected cells. Following a validation screening, the two best inhibitor candidates were N-phenylpyridine-3-carboxamide (NPP3C) and 6-acetyl-1H-indazole (6A1HI). The half maximal effective concentration of NPP3C and 6A1H1 against DENV were 7.1 μM and 6.5 μM, respectively. 6A1H1 decreased infectious DENV particle production up to 1,000-fold without any cytotoxicity at the used concentrations. While 6A1HI was DENV-specific, NPP3C also inhibited the replication of other flaviviruses such as West Nile virus and Zika virus. Structure-activity relationship (SAR) studies with 151 analogues revealed key structural elements of NPP3C and 6A1HI required for their antiviral activity. Time-of-drug-addition experiments identified a postentry step as a target of these compounds. Consistently, using a DENV subgenomic replicon, we demonstrated that these compounds specifically impede the viral RNA replication step and exhibit a high genetic barrier-to-resistance. In contrast, viral RNA translation and the *de novo* biogenesis of DENV replication organelles were not affected. Overall, our data unveil NPP3C and 6A1H1 as novel DENV inhibitors. The information revealed by our SAR studies will help chemically optimize NPP3C and 6A1H1 in order to improve their anti-flaviviral potency and to challenge them in *in vivo* models.

## INTRODUCTION

With approximately 100 million symptomatic infections annually, dengue virus (DENV) poses a major public health challenge worldwide since it causes the most prevalent arboviral disease. Forty percent of the world population (mostly in tropical and subtropical areas) is at risk of becoming infected by DENV, which is transmitted through the bite of carrying Aedes aegypti or Aedes albopictus female mosquitoes. Moreover, presumably because of global warming, these insects are colonizing new territories with more temperate climates, which supports that DENV prevalence will increase in the next decades. DENV can cause severe symptoms in infected individuals such as hemorrhagic fever and shock syndrome, which in some cases may lead to death (1, 2). A tetravalent dengue vaccine is approved in several countries. However, its efficacy is limited depending on the DENV serotype and it is not recommended for seronegative individuals (3). Much effort has been deployed in the last decade to engineer DENV antivirals, but only a few are currently in preclinical development or being challenged in clinical trials (4–6), and still no antiviral therapy is currently available. Hence, there is a medical need to develop new classes of DENV inhibitors to diversify the future therapeutic arsenal, which most probably will involve therapies combining several direct-acting antivirals.

DENV is an enveloped positive-strand RNA virus that belongs to the genus *Flavivirus* within the *Flaviviridae* virus family. Following virus entry by endocytosis, the viral RNA genome (vRNA) is uncoated and translated into a single large viral polyprotein. It is cotranslationally cleaved by viral and host proteases, generating 10 mature viral proteins. The nonstructural proteins NS1, NS2A, NS2B, NS3, NS4A, NS4B, and NS5 are responsible for vRNA synthesis and amplification within membranous structures named vesicle packets (VP), which result from the invagination of the endoplasmic reticulum (ER). The structural proteins capsid (C), premembrane (prM), and envelope (E), together with vRNA, drive viral particle assembly through budding into the lumen of the ER. The virions accumulate in the ER and are released outside the cell through the secretory pathway (7–9).

In this study, we have performed a medium throughput screening of an in-house fragment-based small molecule library in order to identify novel DENV inhibitors. The two identified candidates N-phenylpyridine-3-carboxamide (NPP3C) and 6-acetyl-1H-indazole (6A1HI) were highly efficient to inhibit DENV replication in cell culture. Taking advantage of several cell culture systems based on modified DENV genomes, we have determined that these compounds specifically target the vRNA replication step of the life cycle without altering the biogenesis of VPs. Structure-activity relationship studies with approximately 150 analogues identified key features of both early leads that are absolutely required for their respective antiviral activity. Given that NPP3C and 6A1H1 did exhibit a high barrier to resistance in cell culture, our study identifies these compounds as promising novel DENV inhibitors.

## RESULTS

### A medium-throughput screening identifies NPP3C and 6A1HI as DENV inhibitors.

**FIG 1:**
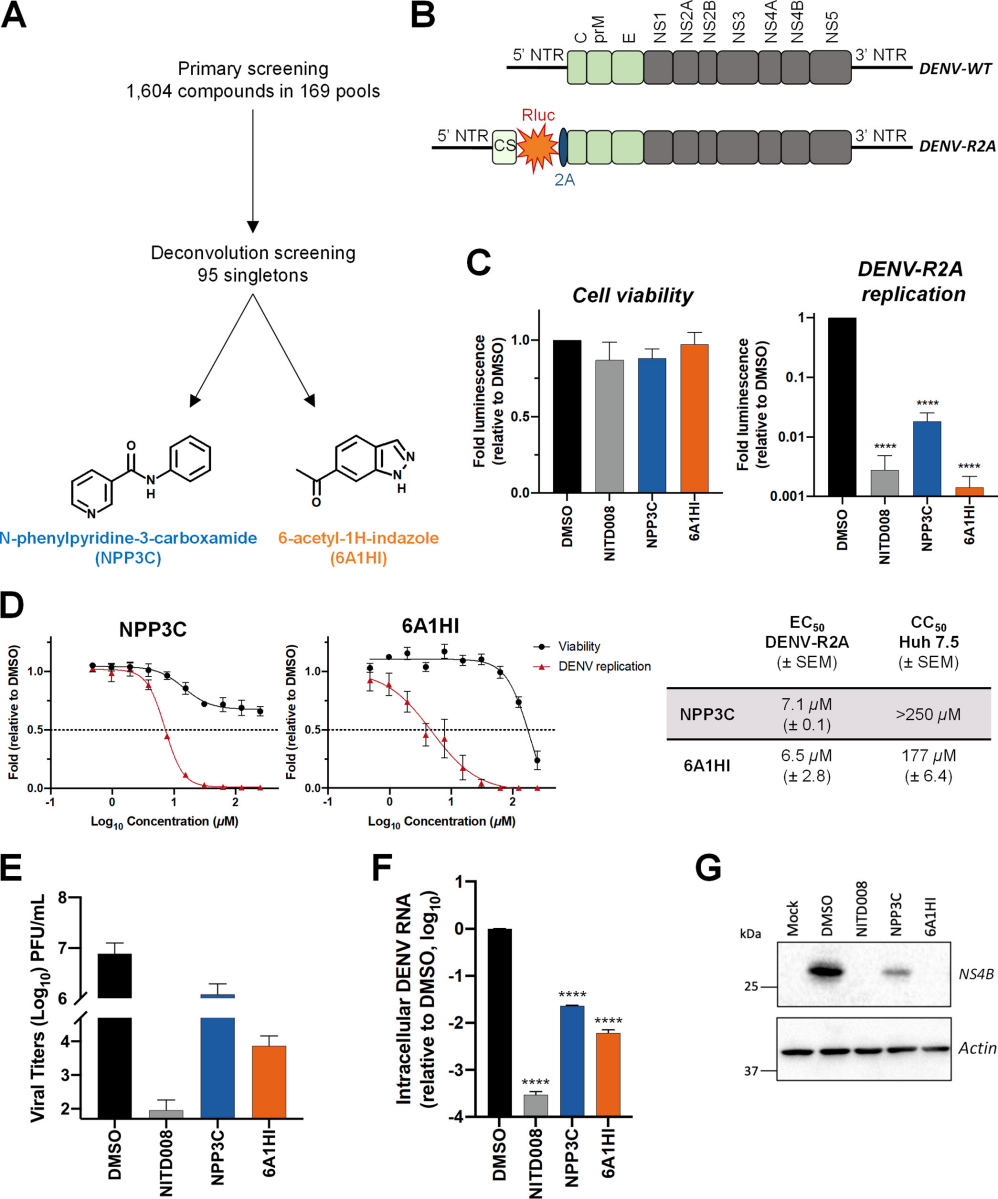
N-phenylpyridine-3-carboxamide and 6-acetyl-1H-indazole inhibit dengue virus replication. (A) The screening pipeline to identify novel DENV inhibitors. The chemical structures of N-phenylpyridine-3-carboxamide (NPP3C) and 6-acetyl-1H-indazole (6A1HI) are shown. (B) Schematic representation of DENV2 16681s (DENV-WT) and DENV2 16681s Renilla-luciferase expressing reporter virus (DENV-R2A) genomes (12). NTR, nontranslated region; CS, 5′ cyclization sequence; 2A, auto-proteolytic foot-mouth disease virus 2A peptide. (C, D) Huh7.5 cells were infected with DENV-R2A (MOI = 0.001) or left uninfected. At 4 h postinfection, cells were treated with NPP3C or 6A1HI at (C) 50 μM (*n* = 4) or (D) at different concentrations (*n* = 3). NITD008 (10 μM) was used as positive control and DMSO as reference control. 2 days postinfection, cell viability was assessed in uninfected cells by CellTiter-Glo luminescent assay. Viral replication was quantified in DENV-R2A infected cells by measuring luminescence. Displayed data are relative to the DMSO treatment. (E–G) Huh7.5 cells were infected with DENV2 16681s at an MOI of 0.1. 2 h postinfection, cells were treated with DMSO, NITD008 (5 μM), NPP3C (50 μM) or 6A1HI (50 μM). Two days postinfection, supernatants were harvested for infectious viral titer quantification by plaque assays (E). Cells were lysed to quantity intracellular viral RNA by RT-qPCR (F) and NS4B viral protein expression by western blotting (G). All the experiments were performed at least three times independently. SEM,: standard error of the mean. ****, *P*-value ≤ 0.0001.

To identify novel DENV inhibitors, we have screened a fragment-based small molecule library that was generated in-house and composed of 169 pools of 7 to 12 molecules (1,604 compounds in total) (Fig. 1A) (10, 11). This library is commercially unavailable, chemically diverse, reliable (i.e., quality-controlled and curated in terms of structure, purity, and solubility, and free-state behavior), and constituted of small fragments likely to show good ligand efficiency upon binding, and suitable for elaboration into higher potency compounds with greater selectivity when required. Huh7.5 hepatocarcinoma cells were infected with a reporter *Renilla reniformis* (Rluc)-expressing luciferase DENV (DENV-R2A, genetically engineered based on serotype 2 strain 16681; Fig. 1B) (12) at a low multiplicity of infection (MOI ~ 0.001) and treated with 150 μM each pool of compounds. Two days later, Rluc activity was measured and compared with that in DMSO-treated cells. In this viral system, the Rluc is produced from the same coding sequence as the viral polyprotein. Hence, the luciferase activity in infected cells is directly proportional to the levels of viral replication. This allowed miniaturization of this sensitive DENV replication assay and a rapid screening of the fragment library with a robust endpoint read-out. In parallel, viability of treated cells was also measured at the same time point to exclude pools that were highly cytotoxic from the analysis. After the primary screening (Table S1), we have selected 10 compound pools for a secondary screening, which individually assessed the anti-DENV activity of 95 singletons initially present in these pools at a concentration of 50 μM. This deconvolution screening identified NPP3C and 6A1HI as primary hits (Fig. 1A and Fig. S1B, Table S2). Indeed, when infected cells were treated with 50 μM NPP3C or 6A1HI, viral replication was decreased 55 and 714-fold, respectively (Fig. 1C) while cell viability was unaffected at this concentration. As an internal control, treatment with NITD008, a specific inhibitor of flaviviral NS5 polymerase (13), potently inhibited DENV replication. To evaluate the antiviral potency of these compounds, we have performed dose-response replication assays by testing 10 compound concentrations in the same infection set-up as that of the screenings. We have determined that the half maximal effective concentration (EC_50_) of NPP3C and 6A1HI were 7.1 μM (±0.1) and 6.5 μM (±2.8), respectively (Fig. 1D). The 50% cytotoxic concentration (CC_50_) of both compounds was above 150 μM, demonstrating a good selectivity index. To confirm the antiviral activity of NPP3C and 6A1HI in DENV life cycle, we measured the production of infectious viral particles from cells infected with wild-type DENV2 (strain 16681s) by plaque assays. The abundance of released virions was reduced upon treatment, especially with 6A1HI with an approximate decrease of 3 Log_10_-fold (Fig. 1E). This correlated with a decrease in the intracellular levels of DENV vRNA and NS4B (Fig. 1F–G). Overall, these data demonstrate that both NPP3C and 6A1HI impede the DENV life cycle.

### Evaluation of NPP3C and 6A1HI broad-spectrum antiviral activity.

**FIG 2:**
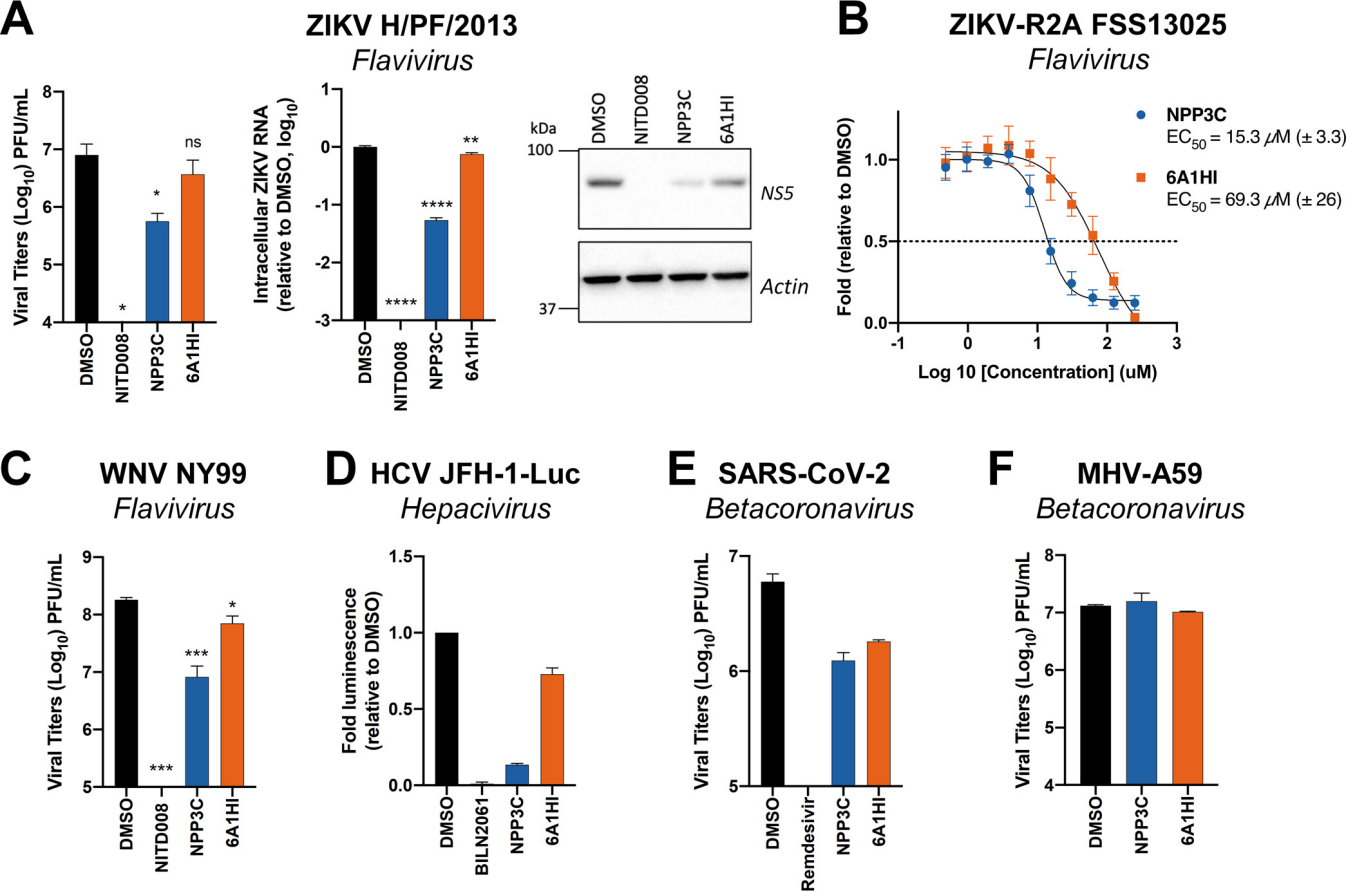
Assessment of NPP3C and 6A1HI broad-spectrum antiviral activity. (A) Huh7.5 cells were infected with ZIKV H/PF/2013 at an MOI of 0.1. Two hours postinfection, cells were treated with DMSO, NITD008 (5 μM), NPP3C (50 μM) or 6A1HI (50 μM). Two days postinfection, supernatants were subjected to plaque assays and intracellular viral replication was assessed by quantification of intracellular viral RNA by RT-qPCR and detection of NS5 viral protein expression by western blotting. ****, *P*-value ≤ 0.0001; **, *P*-value ≤ 0.01; *, *P*-value ≤ 0.05; ns, nonsignificant (*n* = 3). (B) Huh7.5 cells were infected with ZIKV-R2A (MOI = 0.001), treated at 4 h postinfection with NPP3C or 6A1HI at different concentrations. Two days postinfection, viral replication was measured by measuring luminescence and EC_50_ values (± SEM) were determined based on three independent experiments. Displayed data are relative to the DMSO treatment. (C) Huh7.5 cells were infected with WNV NY99 at an MOI of 0.1. Two hours postinfection, cells were treated as in A. Two days postinfection, extracellular infectious titers were determined by plaque assays. ***, *P*-value ≤ 0.001; *, *P*-value ≤ 0.05 (*n* = 3). (D) Huh7.5 cells were electroporated with JFH-1-Luc RNA and 2 h posttransfection, cells were treated as in A. Two days later, viral replication was measured by quantifying firefly luciferase activity. Treatment with 1 μM the HCV NS3 protease inhibitor BILN2061 was used as positive control. (E) Huh7.5 cells were infected with SARS-CoV-2 at an MOI of 0.1. Two hours postinfection, cells were treated as in A. Remdesivir (1 μM) was used as positive control. 2 days postinfection, extracellular infectious titers were determined by plaque assays. (F) DBT cells were infected with MHV-A59 (MOI = 0.0001). One hour postinfection, the viral inoculum was removed, and cells were treated as in A. One day postinfection, supernatants were collected to measure infectious viral titers by plaque assays. Results shown in D–F are representative of two independent experiments.

We next assessed whether NPP3C and 6A1HI exhibit panviral inhibitory activities. To that end, we tested a panel of positive-strand RNA viruses, including other *Flaviviridae* from the *Flavivirus* (Zika virus [ZIKV], strain H/PF/2013, and strain FSS13025 of the Asian lineage; West Nile virus [WNV], strain NY99) and the *Hepacivirus* (hepatitis C virus; HCV, strain JFH-1) genera, as well as two viruses from the *Betacoronavirus* genus in the *Coronaviridae* family, namely, severe acute respiratory syndrome coronavirus 2 (SARS-CoV-2) and mouse hepatitis virus (MHV-A59). Compared to DENV (Fig. 1E-G), ZIKV replication was also impaired to a similar extent when cells were treated with NPP3C (Fig. 2A) with an approximate 15-fold reduction in viral titers. Consistently, the calculated NPP3C EC_50_ value of 15.3 μM (±3.3) against a ZIKV reporter virus (strain FSS13025; ZIKV-R2A) (14) was comparable to that against DENV-R2A (Fig. 2B). In stark contrast, while 6A1HI treatment reduced DENV titers by more than 100-fold, it was much less active against ZIKV (2-fold decrease), which was reflected by a higher EC_50_ value against ZIKV-R2A (69.3 μM [±26] versus 6.5 μM against DENV-R2A) (Fig. 2A-B). Similarly, NPP3C decreased WNV extracellular infectious titers by 22-fold (Fig. 2C). Only moderate impacts (if any) of 6A1HI treatment on the replication of WNV, HCV, and betacoronaviruses were observed (Fig. 2C-F). This strongly supports that 6A1HI antiviral activity is DENV-specific. In contrast, NPP3C treatment did inhibit ZIKV and HCV replication to similar extents, suggesting that it is active against viruses from the *Flaviviridae* family beyond the *Flavivirus* genus. Overall, our results suggest that 6A1HI antiviral activity is selective for DENV while NPP3C inhibits the replication of a broader spectrum of viruses in the *Flaviviridae* family.

### Identification of NPP3C and 6AHI determinants for their antiviral activity.

To identify the structural features of NPP3C and 6A1HI that are required for their antiviral potency, we performed a structure-activity relationship study. We simultaneously tested 70 and 81 commercially available analogues of NPP3C and 6A1HI, respectively, for their capacity to inhibit DENV-R2A at a concentration of 50 μM in the same experimental set-up as that of the deconvolution screen and EC_50_ assays (Fig. 1D and Fig. S1B). Two days postinfection, viral replication and cell viability were measured by luciferase assays and viability assays, respectively. NPP3C and 6A1HI treatments were included as internal positive controls of replication impairment (Tables S3 and S4). These focused screenings revealed critical structural determinants of NPP3C and 6A1HI antiviral activity that are illustrated in Tables 1 and 2, respectively. For instance, the substitution of the pyridine group of NPP3C by a phenyl (compound 66) abrogated the antiviral activity. Moreover, any analogue of NPP3C with a modified central carboxamide scaffold (compounds 54, 63 and 56) retains little activity, if any. Similar phenotypes were observed upon the introduction of several different substituents on the phenyl moiety of NPP3C. Regarding 6A1HI, the 6-acetyl group plays a key role, as the inhibitory activity was completely lost when the acetyl group was moved to positions 4 or 7 of the indazole (compounds 52 and 9) instead of position 6. Furthermore, the same was observed upon replacement of the 6-acetyl group by other substituents, such as hydroxymethyl, hydroxyl, aminomethyl, and carboxamide (compounds 3, 7, 6, and 75, respectively). Modifications on the indazole core, including N-methylation (compounds 76, 42, and 4), likewise led to a loss of inhibitory activity. To sum up, we identified some key determinants of NPP3C and 6A1HI required for their antiviral potency.

**TABLE 1:** Selected NPP3C analogues from SAR studies

Compound	Structure	DENV-R2A replication (fold DMSO)	Cell viability (fold DMSO)
NPP3C	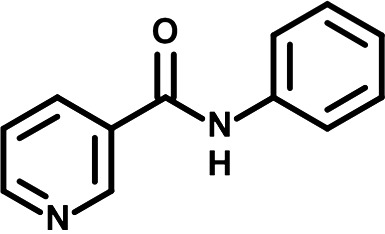	0.01	0.80
66	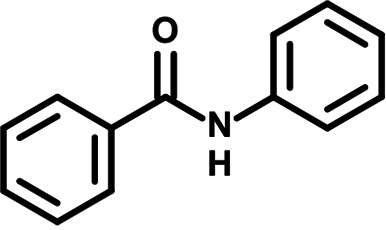	1.34	0.88
7	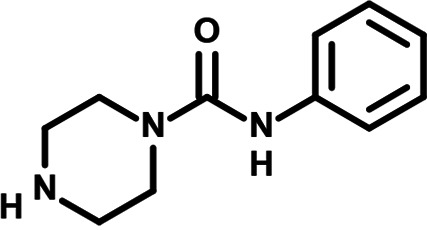	0.82	1.16
29	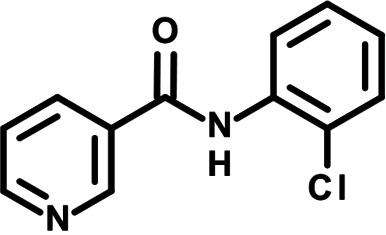	0.99	0.95
65	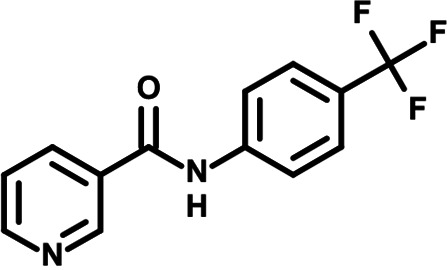	0.98	0.87
55	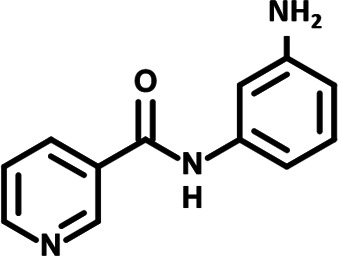	0.75	0.96
52	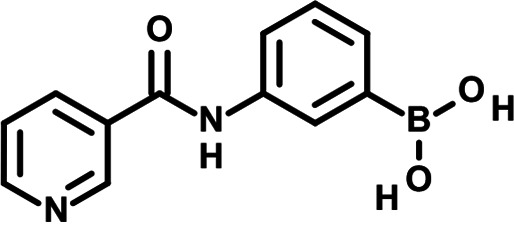	1.07	0.95
54	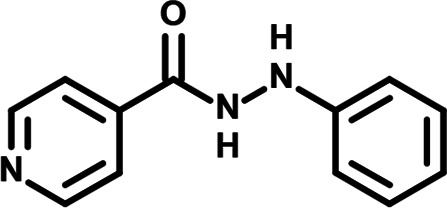	0.37	0.67
63	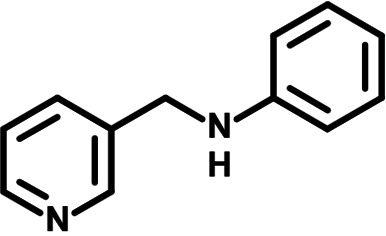	1.38	0.90
56	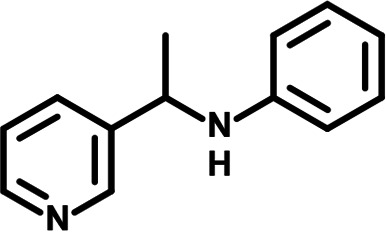	1.47	0.94

**TABLE 2:** Selected 6A1HI analogues from SAR studies

Compound	Structure	DENV-R2A replication (fold DMSO)	Cell viability (fold DMSO)
6A1HI	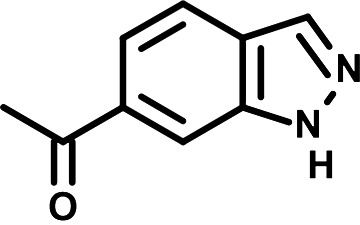	0.04	0.93
3	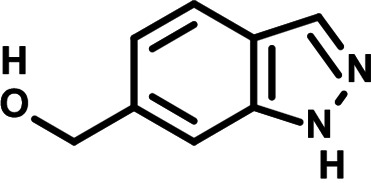	1.10	0.93
7	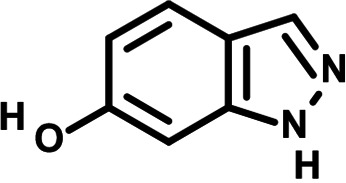	1.05	1.07
18	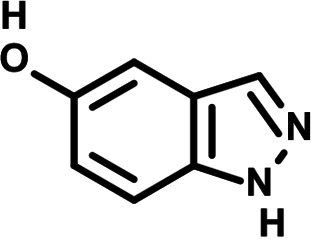	1.06	0.98
6	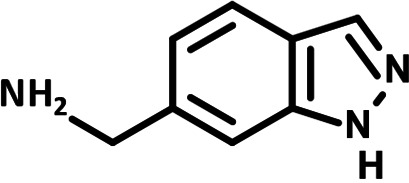	0.96	0.76
75	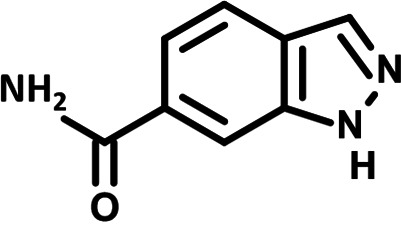	1.06	0.99
9	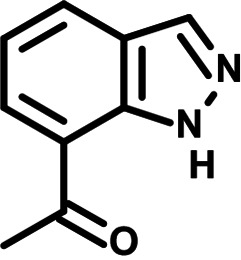	0.90	1.03
52	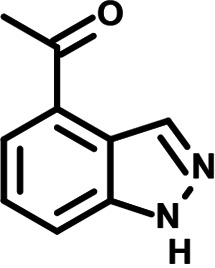	1.15	0.93
76	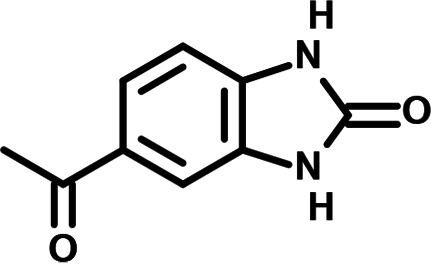	0.87	0.98
4	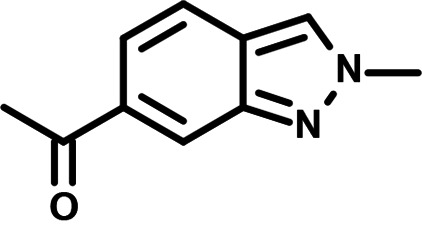	0.97	0.75
42	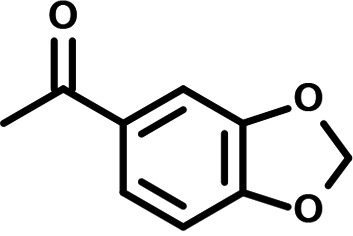	1.07	0.93

### NPP3C and 6A1HI inhibit the RNA synthesis step of DENV life cycle.

**FIG 3:**
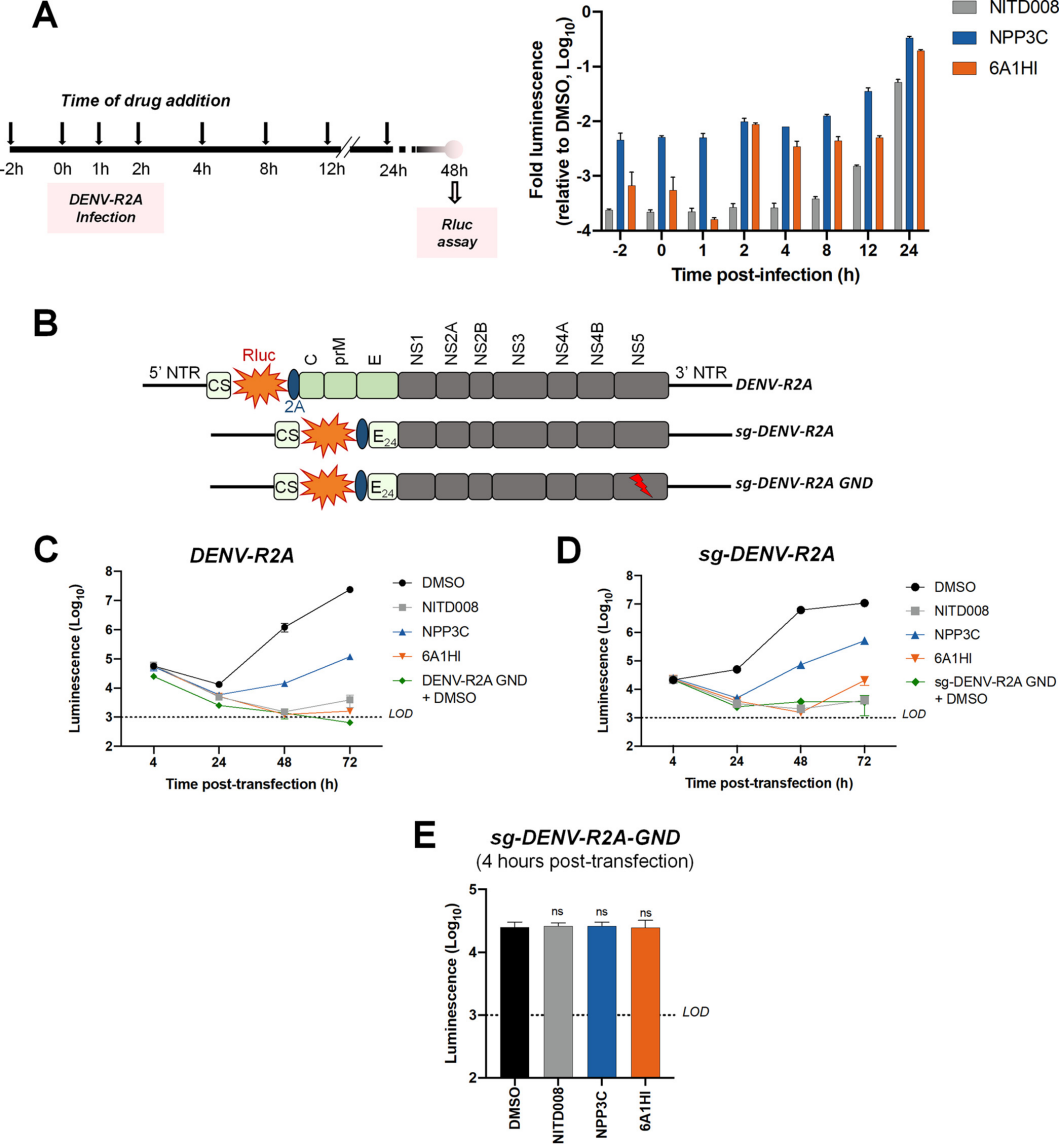
NPP3C and 6A1HI inhibit the viral RNA replication step of DENV life cycle. (A) Huh7.5 were infected with reporter virus DENV-R2A and treated with DMSO (negative control), NITD008 (10 μM, positive control), NPP3C (50 μM), and 6A1HI (50 μM), added at different time points. At 2 days postinfection, Rluc assays were performed to measure viral replication. The results are representative of two independent experiments, each including biological duplicates. (B) Schematic representation of the modified DENV genomes which were electroporated in C–E. (C–E) Huh7.5 were electroporated with *in vitro*-transcribed (C) DENV-R2A full RNA genome, (D) sg-DENV-R2A subgenomic replicon, or (E) sg-DENV-R2A GND replication-incompetent subgenome and immediately treated with DMSO, 50 μM NPP3C, 50 μM 6A1HI, or 10 μM NITD008. At the indicated time points, transfected cells were subjected to Rluc assays. Results shown in C–E are representative of three independent experiments.

We next sought to determine which step of DENV life cycle is targeted by NPP3C and 6A1HI. We first performed antiviral assays in an infection set-up in which the candidate compounds were added at different time points before and after infection with Rluc-encoding DENV reporter viruses (Fig. 3A). Viral replication was measured by luciferase assays 2 days postinfection. Both NPP3C and 6A1HI retained a strong inhibitory activity when they were added up to 8 h after infection, a time point by which virus entry and vRNA uncoating are resolved (15). This strongly suggests that both drugs target a viral process that occurs downstream of these first steps of DENV life cycle, which includes vRNA translation and NS5 polymerase-dependent RNA synthesis.

To determine whether NPP3C and 6A1HI inhibit DENV vRNA synthesis and/or translation, we took advantage of modified DENV genomes (Fig. 3B) (12, 16) and examined the impact of drug treatment on Rluc expression from these *in vitro*-transcribed and electroporated RNAs. As a control, we first assessed that both compounds were still active against DENV when full-length DENV-R2A vRNA was electroporated. In contrast to the replication-defective DENV-R2A GND RNA (containing a point mutation in NS5 coding sequence), wild-type DENV-R2A efficiently replicated in transfected cells over time (Fig. 3C). As expected, when NITD0008 was added 4 h after electroporation, viral replication was almost completely abrogated. Similarly, DENV replication was severely impacted upon treatments with either NPP3C or 6A1HI, confirming their antiviral activity in this RNA transfection set-up. Next, we performed the same experiment using DENV subgenomic replicons expressing Rluc (sg-DENV-R2A). These modified vRNA genomes are replication-competent once introduced into cells by electroporation but lack the coding sequences of structural proteins and, therefore, are unable to produce viral particles (Fig. 3B). However, because they do not express structural protein, no viral particles are produced. Thus, this system only recapitulates the vRNA translation and replication steps of DENV life cycle whose efficiency is measured with luciferase assays. When sg-DENV-R2A-transfected cells were treated with NPP3C and 6A1HI for 2 days, viral replication was decreased by approximately 1.3 and 2.7 Log_10_, respectively (Fig. 3D). As controls, very little replication, if any, was observed when these cells were treated with NITD008 or when a replication incompetent subgenome (sg-DENV-R2A GND) was electroporated. Importantly, since RNA replication, viral particle assembly, and viral entry do not occur when sg-DENV-R2A GND RNA is electroporated, luciferase activity at 4 h posttransfection can be used as a read-out of viral RNA translation (Fig. 3E). Compared to the DMSO control, when cells were treated with NPP3C or 6A1HI, Rluc activity remained unchanged, suggesting that both inhibitors do not inhibit viral RNA translation. Altogether, our data unambiguously demonstrate that NPP3C and 6A1HI specifically target the vRNA synthesis step of DENV life cycle.

To identify the viral protein targeted by NPP3C and 6A1HI and gain insight into their mode-of-action, we attempted to select DENV genomes with mutations that confer resistance to the inhibitors. To that aim, we took advantage of selectable DENV subgenomic replicons that encode the hygromycin phosphotransferase gene (Fig. S2A) (12). With that system, only cells containing a replicating genome can survive upon hygromycin B treatment (Fig. S2B, top left panel). In the presence of hygromycin B and an inhibitor of interest, it is expected that only genomes that are resistant to the latter drug will replicate and confer survival to the cell. In that context, once resistant clones are isolated, vRNA can be extracted and sequenced to identify the mutated viral protein(s). Remarkably, after 31 days of dual selection, we were unable to select resistant cells (Fig. S2B). Hence, while we were unable to identify the viral protein(s) targeted by NPP3C and 6A1H1 with this approach, this suggests that these two DENV replication inhibitors exhibit a high genetic barrier to resistance.

### NPP3C and 6A1HI do not impede the biogenesis of DENV vesicle packets.

**FIG 4:**
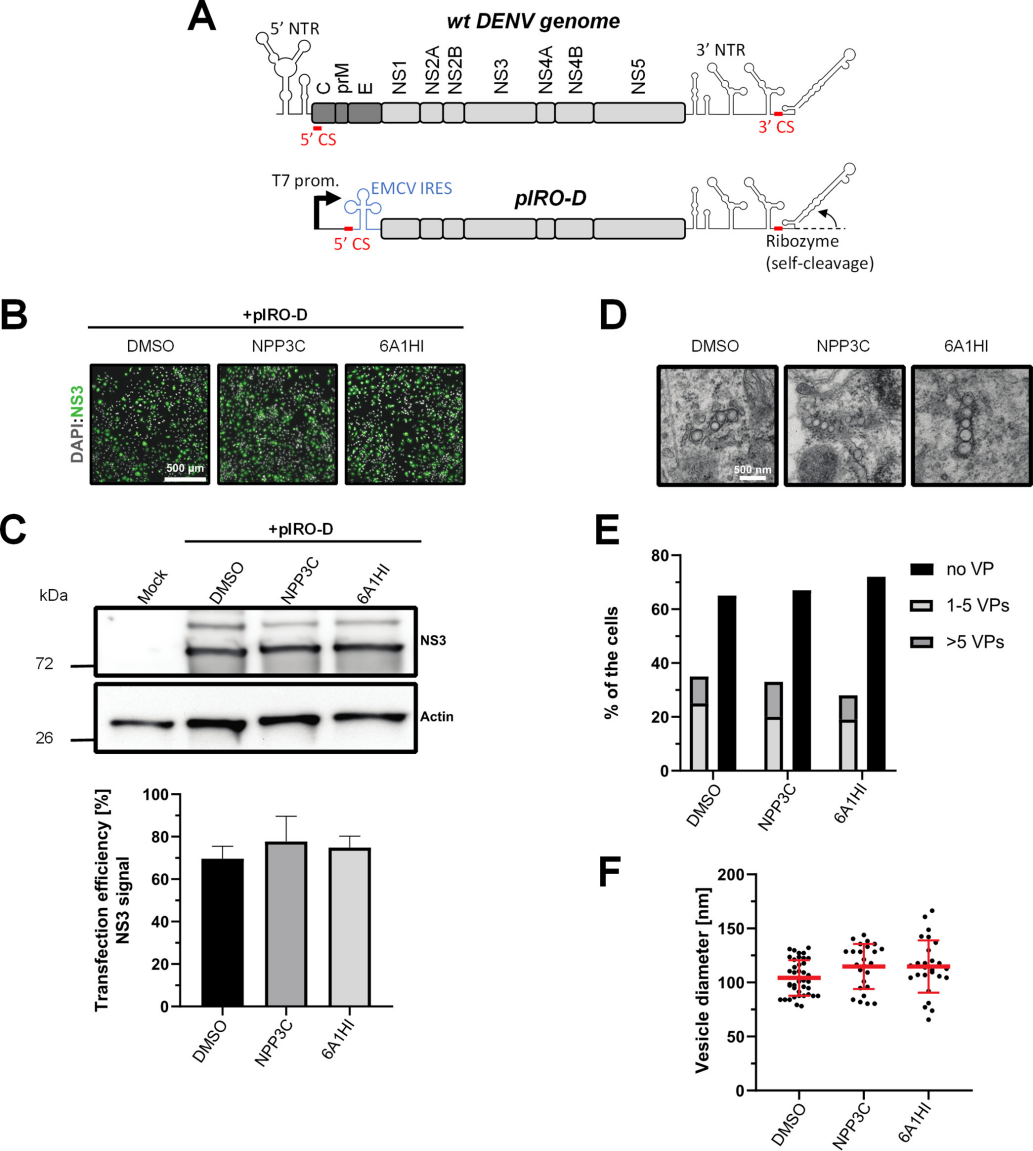
The *de novo* formation of DENV replication organelles is not impacted by NPP3C or 6A1HI treatment. (A) Schematic representation of DENV genome and pIRO-D plasmid. NTR: nontranslated region; CS: cyclization sequence; EMCV IRES: Internal ribosome entry site of encephalomyocarditis virus. (B–F) Huh7/Lunet-T7 cells were transfected with pIRO-D plasmid and treated for 12 h with DMSO, 50 μM NPP3C, or 50 μM 6A1HI. 16 h posttransfection, cells were analyzed for immunofluorescence, western blot and electron microscopy. (B) Transfection efficiency upon treatment with the different compounds was evaluated by immunostaining with anti-NS3 antibody. (C) Total lysates of transfected and treated cells were analyzed by western blot using the indicated antibodies. (D) Electron microscopy analysis of samples treated as in (A). Scale bar: 500 nm (E) Quantification of the number of cells showing vesicle packets. At least 15 cells from two different experiments have been analyzed for each condition. (F) Measurement of VP diameter.

Flaviviral vRNA synthesis relies on the formation of replication organelles. Indeed, VPs, resulting from the invagination of ER membranes contain nonstructural proteins as well as double-stranded RNA, the viral RNA replication intermediate. Since NPP3C and 6A1HI inhibit vRNA replication, we hypothesized that this could be mediated by an alteration of VP morphogenesis upon treatment. To specifically test this, we took advantage of a recently described plasmid transfection-based system (pIRO-D) that allows *de novo* formation of VPs in transfected cells in a replication-independent manner (Fig. 4A) (17–19). In this system, the DENV NS1-5 polyprotein is expressed under the control of a T7 promoter and an internal ribosome entry site (IRES) following transfection of Huh7-derived Lunet cells expressing the T7 RNA polymerase (Huh7/Lunet-T7). RNAs generated in transfected cells contain the DENV 5′ CS and 3′ nontranslated region, the latter followed by a ribozyme to generate correct 3′ ends of these RNAs. The viral proteins generated from these RNAs induce VPs with a genuine architecture in the absence of viral genome replication. pIRO-D plasmid-transfected Huh7/Lunet-T7 cells were treated with NPP3C and 6A1HI and analyzed by western blotting and microscopy. To evaluate whether the inhibitors alter VP biogenesis, we first showed that neither treatment impacted the overall transfection efficiency as determined using immunofluorescence microscopy (Fig. 4B). Moreover, western blot analysis demonstrated that the levels of viral protein NS3 remained unchanged (Fig. 4C) upon treatment, showing that the inhibitors do not alter polyprotein expression and cleavage. The analysis of transfected cells by transmission electron microscopy revealed that neither of the treatments impacted the abundance of VPs or their size and shape (Fig. 4D-F). Overall, these data show that NPP3C and 6A1HI do not inhibit the *de novo* formation of DENV VPs. Thus, these compounds target vRNA synthesis factors or processes that are unrelated to the morphogenesis of DENV replication organelles.

## DISCUSSION

In this study, following a phenotypic medium-throughput screen of a fragment-based library, we have identified NPP3C and 6A1HI as inhibitors of DENV life cycle. 6A1HI activity was mostly DENV-specific while NPP3C exhibited broader antiviral properties since treatment with this compound negatively affected WNV, ZIKV, and HCV replication. Interestingly, both compounds had a modest anti-SARS-CoV-2 activity (less than 10-fold reduction) but did not impede the replication of MHV, another betacoronavirus. Compared to other DENV and ZIKV inhibitors that we have recently reported (20), NPP3C showed greater potency than the Amaryllidaceae alkaloid cherylline when tested in the same experimental set-up (EC_50_ DENV = 8.8 μM; EC_50_ ZIKV = 20.3 μM; CC_50_ > 250 μM) as the one used here. The subsequent screening of 151 commercially available analogues of NPP3C and 6A1HI revealed key chemical features of these compounds to maintain their antiviral activity (Tables 1 and 2), especially regarding the importance of NPP3C central carboxamide core and of the acetyl group’s position in 6A1HI. These SAR studies will be extended in the next phase of research, including the search for significant improvements in potency by expanding and testing much larger compound collections. Fortunately, many related analogues are commercially available, and the synthetic schemes of the leads are relatively simple. The collected information about NPP3C and 6A1HI structural restraints will be pivotal for further synthetic chemistry-driven optimization in future SAR studies to improve their antiviral potency. For instance, it will be relevant to explore whether the addition of lateral chains or other functional groups to the pyridine moiety of NPP3C improves its antiviral potency. Alternative options would be to chemically link compounds from each series to see if additive or synergistic potency improvements are observed, or to merge them to identify a novel scaffold with potential further gains in potency.

Taking advantage of reporter subgenomic replicons, we have demonstrated that these two compounds primarily target the vRNA replication step. Consistently, time-of-drug-addition experiments allowed us to rule out an inhibition of viral entry, fusion, and genome uncoating since NPP3C and 6A1HI retain their full inhibitory activity when added 4 h after infection, a time point at which these processes are resolved (15). The replication of DENV RNA occurs within ER-derived VP with the contribution of all nonstructural proteins, including NS5 RNA polymerase (7–9, 17). Evaluating a potential direct inhibition on VP biogenesis is challenging because any decrease in replication (even if unrelated to VPs) will inevitably impact the expression of viral proteins and, thus, VP formation. To tackle this challenge, we used a replication-independent DENV replication organelle formation system (18, 19) and demonstrated that NPP3C and 6A1HI treatments did not impact the abundance, size, and shape of DENV VPs, ruling out that these compounds inhibit VP morphogenesis and presumably, specific activities of cellular or viral proteins regulating this process (e.g., NS4A, ATL2, RTN3) (21–25). Consistently, drug treatments did not affect NS3 levels and self-cleavage when expressed as a polyprotein (Fig. 4C) or as a NS2B-3 precursor (unpublished data), demonstrating that NPP3C and 6A1HI do not target NS3 protease activity. Coimmunoprecipitation assays also showed that these compounds do not inhibit the formation and stability of ZIKV NS3/NS4B interaction (unpublished data), which, in case of DENV, is important for RNA replication and is disrupted by JNJ-A07, a highly potent anti-DENV inhibitor (4, 16, 26). Thus, the modes-of-action of NPP3C and 6A1HI likely differ from the one described for JNJ-A07. Considering all this, it is possible that these compounds inhibit one of DENV NS5 enzymatic functions such as its RNA polymerase activity. It is noteworthy that Cheng and colleagues reported that several pyridine carboxamides with structures more complex than that of NPP3C, inhibit NS5B RNA polymerase of hepatitis C virus in the micromolar range most likely through the binding at the palm site motif on NS5B (27). Considering the structural similarity between the indazole moiety of 6A1HI and purines, it is tantalizing to speculate that this compound is a nucleotide inhibitor, even if it was DENV-specific. It will be important to assess whether NPP3C and 6A1HI inhibit NS5 RNA polymerase activity in *in vitro* enzymatic assays.

In an effort to identify the viral protein(s) putatively targeted by NPP3C and 6A1HI, our attempts to isolate resistant DENV genomes using selectable replicons were unsuccessful. This suggests that these inhibitors exhibit a high genetic barrier to resistance, a significant asset when considering preclinical development. One explanation for this apparent incapacity to generate resistance could be that NPP3C and 6A1HI do not directly target viral proteins but instead target host factors that are involved in cellular processes critical for viral replication. Such inhibition might be harder to be by-passed by DENV via compensatory or replication enhancing mutations in DENV proteins. There are no reported cellular targets of NPP3C and 6A1HI. However, illustrating this possibility, it is noteworthy to mention that several complex phenylcarboxamide-containing compounds were reported to inhibit cellular proteins such as PD-L1, FOXM1 and GAK (28–30). Interestingly, the treatment with the antitumoral compound N-(2-[aminocarbonyl]phenyl) (1,1′-biphenyl)-4-carboxamide was effective in an *in vivo* murine cancer model without observable toxicity (30), supporting that phenylcarboxamides are generally safe and bioavailable. Considering that 6A1HI is more active against DENV than ZIKV, this hypothetical scenario of a 6A1HI-targeted host factor would highlight a specific dependency of DENV replication on the inhibited cellular machinery. To clearly determine the mode-of-action of NPP3C and 6A1HI, future studies will investigate to which viral or cellular proteins these inhibitors bind. An alternative strategy would be to select for viral resistance by passaging the wild-type virus using dose-escalation of the drug over time instead of the high drug pressure that we applied in this study with the use of selectable DENV replicons. Additionally, a more direct approach for viral or host target identification could involve the synthesis of active biotin-cross-linked compounds. After treatment of cells infected with DENV or expressing the viral polyprotein (as in Fig. 4) with these modified drugs generated, biotinylated drug-associated proteins could be purified using a streptavidin-coupled resin and subsequently identified by liquid chromatography-mass spectrometry. One challenge associated with such an approach will be to retain the antiviral activity of the leads upon biotinylation. In that respect, our SAR studies reported here are highly informative about the limitations associated with NPP3C and 6A1HI modifications. Finally, it will be highly relevant to evaluate the pharmacological potential of NPP3C and 6A1HI antiviral activity (or that of more active derivatives) *in vivo* using murine DENV infection models, warranting further preclinical development of these promising compounds.

## MATERIALS AND METHODS

### Cell lines.

RIG-I-deficient human hepatocarcinoma cell line Huh7.5 and Vero E6 cells were cultured in DMEM (Life Technologies) containing 10% fetal bovine serum (FBS; Wisent), 1% penicillin-streptomycin (PS; Thermo-Fisher) and 1% nonessential amino acids (NEAA; Thermo-Fisher). Huh7/Lunet-T7, described in (18), were cultured in DMEM-10%FBS-1%PS-1%NEAA supplemented with 5 μg/mL zeocin (Invitrogen). HRT-18 cells were cultured in AMEM (Wisent) containing 10% FBS, 1% PS and 1% NEAA. DBT (delayed brain tumor) cells were cultured in EMEM (Wisent) containing 10% FBS (PAA) and 1 mM sodium pyruvate (Invitrogen). All cells were maintained at 37°C and 5% CO_2_.

### Viruses.

Plasmids encoding flavivirus genomes sequences coding for *Renilla* luciferase (pFK-DENV-R2A, pFL-ZIKV-R2A), reporter subgenomic-replicon (pFK-sgDENV-R2A), and replication-incompetent (pFK-sg-DENV-R2A GND) and for selectable subgenomic replicon (pFK-sg-DENV-R2H) and for pTM/DENV Δ5′ SLAB-3′ wt-Ribozyme (pIRO-D) were previously described (12, 14, 16). Plasmids were linearized using XbaI (DENV) or ClaI (ZIKV). *In vitro* transcription was then performed on linearized plasmids using the mMessage mMachine kit (Thermo-Fisher) with T7 (ZIKV) or SP6 (DENV) polymerase.

A replicative bicistronic JFH1-based full genome, expressing Firefly luciferase (pJFH1/Fluc) was produced as previously described (31). Briefly, pJFH1/Fluc plasmid was linearized with XbaI prior to purification with DNA Clean & Concentrator (Zymo Research). *In vitro* transcriptions were performed using T7 Ribomax Express Large Scale RNA Production System (Promega) according to the manufacturer’s instructions. The quality of the *in vitro* transcripts was controlled by electrophoresis on an agarose gel.

DENV2 16681s and Rluc-expressing reporter viruses (DENV-R2A and ZIKV-R2A) were produced after electroporation of *in vitro* transcribed genomes in Vero E6 cells. Briefly, confluent Vero E6 were trypsinized and resuspended in complete DMEM. The cells were washed with PBS and resuspended in a Cytomix buffer (120 mM KCl, 0.15 mM CaCl_2_, 10 mM potassium phosphate buffer [pH 7.6], 25 mM HEPES [pH 7.6], 2 mM EGTA, 5 mM MgCl_2_ [pH 7.6], freshly supplemented with 2 mM ATP, and 5 mM glutathione) at a density of 1.5 × 10^7^ cells per mL. 400 μL of cell suspension were mixed with 10 μg of *in vitro* transcribed RNA. The cells were then transferred to an electroporation cuvette (0.4 cm gap width; Bio-Rad) and pulsed with a Gene Pulser Xcell Total System (Bio-Rad) at 975 μF and 270 V. Cells were then seeded in a 15 cm petri dish. One day after electroporation, cell culture medium was replaced with complete DMEM. Supernatants containing viruses were harvested from day 4 to 7 posttransfection, filtered through a 0.45 μm syringe filter, and supplemented with 10 mM HEPES (pH 7.5). Virus aliquots were stored at −80°C and infectious viral titers were determined by plaque assay exactly as previously described (17, 32, 33).

ZIKV H/PF/2013 and WNV NY99 strains were obtained from European Virus Archive Global (EVAg) and were grown in Vero E6 cells exactly as previously described (33, 34). Stocks of SARS-CoV-2 (strain hCoV-19/Canada/ON-VIDO-01/2020|EPI_ISL_425177|2020-01-23 [35]) were produced in Vero E6 cells. The viral titers were determined by plaque assays in Vero E6 cells.

MHV-A59 was kindly provided by Pierre Talbot and was passaged on DBT cells (MOI = 0.0001). Viral titers were determined on DBT cells by standard plaque assays, with modifications to the previously described method (36). Briefly, 24-well plates were preseeded with DBT cells to obtain ~5 × 10^5^ cells/well at time of infection. Serially diluted samples were incubated for 1 h with cell monolayers and inoculums replaced with overlay (MEM-Earle (Invitrogen) supplemented with 2% methylcellulose (Sigma), 1% FBS (PAA), 2 mM l-glutamine (Invitrogen), 0.13% sodium bicarbonate (Wisent), and 1% PS (Wisent). After 48 h, the overlay was removed and cells were fixed and stained (0.5% [wt/vo]) crystal violet in a 50% ethanol, 1.85% formaldehyde, and 0.8% (wt/vol) sodium chloride solution) for plaques counting.

### Fragment-based compound library screening.

The library of fragment-based compounds was previously described (10, 11) and kindly provided by NMX Research and Solutions Inc. Briefly, it was composed of 169 pools containing 7 to 12 compounds per pool, for a total of 1,604 compounds (all originally sourced from Key Organics). For the primary screen, the antiviral activity of each fragment was assessed at 150 μM in DMSO. Pools with cytotoxicity lower than 20% and anti-DENV and anti-ZIKV activities higher than 50% were selected for deconvolution screen. During the deconvolution screen, each compound was tested at 50 μM.

2 × 10^6^ Huh7.5 cells were seeded in 10 cm dishes and cultured overnight. The following day, they were infected with DENV-R2A or ZIKV-R2A (MOI = 0.005). Virus inoculum was removed 4 h later and cells were washed with PBS, trypsinized, and seeded in 96-well plate with various concentrations of compounds. DMSO and NITD008 (Tocris Small Molecules) were used as reference and positive control, respectively. Two days later, cells were prepared for Rluc assays.

### Luciferase assays.

DENV-R2A/ZIKV-R2A-Infected cells were lysed in 100 μL lysis buffer (0.1% Triton X-100, 25 mM glycylglycine [pH 7.8], 15 mM MgSO_4_, 4 mM EGTA [pH 8], and 1 mM DTT). RLuc assay was performed by mixing 30 μL of the lysate with 150 μL of assay buffer (25 mM glycylglycine [pH 7.8], 15 mM MgSO_4_, 4 mM EGTA [pH 8], 15 mM K2PO_4_ [pH 7.8]) and coelenterazine (1.43 μM, Prolume). Luminescence was measured using Spark multimode microplate reader (Tecan).

To assess the efficiency of drugs on HCV, Huh7.5 cells were washed twice in PBS, resuspended in Ingeno buffer (Mirus) at 1 × 10^7^ cells/mL. Electroporation was performed in 0.2 cm cuvette with 2 × 10^6^ cells using the following parameter: 820 V, 99 μs, 4 pulses, 1.1 s interval with an ECM 830 Electro Square Porator (BTX). Electroporated cells were seeded in 24-well plates with 1.5 × 10^5^/well. Four hours later, medium was changed to complete DMEM media containing NPP3C or 6A1HI at 50 μM. DMSO and BILN2061, a HCV NS3 protease inhibitor (a kind gift from Daniel Lamarre, University of Montreal), were used as negative and positive control, respectively. Cells were lysed and tested for luciferase 48 h posttransfection using 100 μL of BrightGlo reagent (Promega). Firefly luciferase activity was measured with a Spark multimode microplate reader (Tecan). NPP3C, 6A1HI, and their 151 analogues were obtained from Key Organics.

### Cytotoxicity assay.

Cell viability in Huh7.5 cells was measured using CellTiter-Glo assay; 2 × 10^6^ cells were seeded in 10 cm petri dishes and cultured overnight. The following day, cells were trypsinized and 15 × 10^3^ cells were plated in 96-well plates. Compounds were added at indicated concentration for 48 h. Two days posttreatment, 100 μL of room temperature CellTiter-Glo reagent (Promega) was added in each well. Plates were incubated for 10 min and luminescence signal was measured using Spark multimode microplate reader (Tecan). Percentage of viable cells and fold viability were calculated at each concentration by comparing values to DMSO-treated cells.

### Western blot analysis.

Infected Huh7.5 cells were washed twice with cold PBS and lysed in lysis buffer (0.5% Triton X-100, 150 mM NaCl, 50 mM Tris [pH 7.8], and EDTA-free protease inhibitors [Roche]). Samples were denatured by incubating them at 95°C for 5 min. After SDS-PAGE, proteins were blotted onto nitrocellulose membranes that were blocked with 5% milk in PBS-T (PBS containing 1% Tween) for 1 h at room temperature (RT). Membranes were incubated with primary antibodies diluted in 3% BSA in PBS, for 1 h, and washed three times for 10 min each with PBS-T. Horseradish peroxidase (HRP)-conjugated secondary antibodies were diluted in 5% milk in PBS-T, and membranes were incubated for 1 h at RT. After washing three times with PBS-T for 10 min each, membranes were developed by enhanced chemiluminescence. The generation of rat anti-NS3 antibodies was reported previously (32). Rabbit anti-NS4B and anti-NS3 antibodies were obtained from Genetex; Mouse anti-actin antibodies were from Millipore-Sigma.

### RT-qPCR.

Total RNA was extracted from cells using the RNeasy minikit (Qiagen) and subjected to reverse transcription using the Invitrogen SuperScript IV VILO Master Mix RT kit (Life Technologies). Real-time PCR was performed using the Applied Biosystems SYBR green Master mix (Life Technologies) and a LightCycler 96 (Roche) for the detection. The following primer pairs were used: 5′-GCCCTTCTGTTCACACCATT-3′ and 5′-CCACATTTGGGCGTAAGACT-3′ for DENV 16681s; 5′-AGATGAACTGATGGCCGGGC-3′ and 5′-AGGTCCCTTCTGTGGAAATA-3′ for ZIKV H/PF/2013; 5′-GAAGGTGAAGGTCGGAGTC-3′ and 5′-GAAGATGGTGATGGGATTTC-3′ for *GAPDH*. The ΔΔCt method was used to determine the relative expression levels normalized to GAPDH.

### Replication-independent VP induction.

Huh7/Lunet-T7 cells were seeded on glass coverslips and transfected with pTM/DENV Δ5′ SLAB-3′ wt-Ribozyme (pIRO-D) (18) plasmid and TransIT-LT1 transfection reagent (Mirus). Four hours later, the medium was changed, and cells were treated with DMSO or 50 μM NPP3C or 6A1HI for 12 h. Sixteen hours posttransfection, the cells were fixed using an EM fixative (50 mM sodium cacodylate buffer, 50 mM KCl, 2.6 mM MgCl_2_, 2.6 mM CaCl_2_, 1% paraformaldehyde, 2.5% glutaraldehyde, and 2% sucrose [pH 7.4]) and subsequently processed for transmission electron microscopy (TEM) as previously described (17–19). Experiments were conducted in biological duplicates and at least 15 cells were analyzed for presence of VPs for each condition. The diameter of VPs was determined using Fiji image processing package (open-source platform). In parallel to the TEM analysis, cells were also assessed for viral protein expression by Western blot and immunofluorescence analysis using an NS3-specific antibody and Alexa Fluor-conjugated secondary antibodies.

### Statistical analysis.

All Student t-tests were unpaired and two-tailed.
