# Supplementary material for: N-Phenylpyridine-3-Carboxamide and 6-Acetyl-1H-Indazole Inhibit the RNA Replication Step of the Dengue Virus Life Cycle
Source: Antimicrob Agents Chemother. 2023 Jan 26;67(2):e01331-22. doi: 10.1128/aac.01331-22 (PMC9933715; doi:10.1128/aac.01331-22)
Supplement: Supplemental file 5 — Fig. S1 and S2 and legends to Tables S1 to S4. Download aac.01331-22-s0005.pdf, PDF file, 0.3 MB [file aac.01331-22-s0005.pdf]

SUPPLEMENTAL FIGURES LEGENDS

Supplementary Figure 1: A medium throughput phenotypic screening identifies NPP3C and 6A1HI as inhibitors of DENV replication. (A) Schematic representation of DENV2 16681s and ZIKV Renilla-luciferase expressing reporter viruses (DENV-R2A and ZIKV-R2A, respectively) genomes. NTR: non-translated region; CS: 5' cyclization sequence; 2A: auto-proteolytic foot-mouth disease virus 2A peptide. (B-C) Results of the secondary screens of singleton compounds against DENV-R2A (B) and ZIKV-R2A (C). Huh7.5 cells were infected with DENV-R2A or ZIKV-R2A (MOI=0.001) or left uninfected. After 4 hours, cells were treated with one of 95 singleton compounds (50 µM). 2 days post-infection, viral replication was measured by quantifying luminescence and cell viability was assessed in uninfected cells by CellTiter-Glo luminescent assay. Displayed data are relative to the DMSO treatment. NPP3C and 6A1HI are represented in red.

Supplementary Figure 2: NPP3C and 6A1HI exhibit a high genetic barrier to resistance
(A) Schematic representation of sg-DENV-R2H genome. NTR: non-translated region; CS: 5' cyclization sequence; 2A: auto-proteolytic foot-mouth disease virus 2A peptide; HygroR: hygromycin phosphotransferase gene. (B) Huh7.5 cells were electroporated with sg-DENV-R2H RNA or left untransfected. The following day, hygromycin (500 µg/mL) was added to the cell culture medium and cells were treated with DMSO, NITD008 (25 µM), NPP3C (100 µM) or 6A1HI (100 µM). Medium was changed once a week. After 31 days post-electroporation, cells were fixed and stained with 1% crystal violet/10% ethanol. Untransfected parental Huh7.5 cells, treated with hygromycin (500 µg/mL), served as a killing control. The experiment was performed in triplicate. Representative pictures are shown.

Supplementary Table 1: Results of the primary phenotypic screening of the compound pools assessing effects on DENV-R2A replication and cell viability.

Supplementary Table 2: Results of the deconvolution screening of 95 singletons assessing effects on DENV-R2A and ZIKV-R2A replication and cell viability.

Supplementary Table 3: SAR studies with 70 NPP3C analogues assessing effects on DENV-R2A replication and cell viability.

Supplementary Table 4: SAR studies with 81 6A1HI analogues assessing effects on DENV-R2A replication and cell viability.

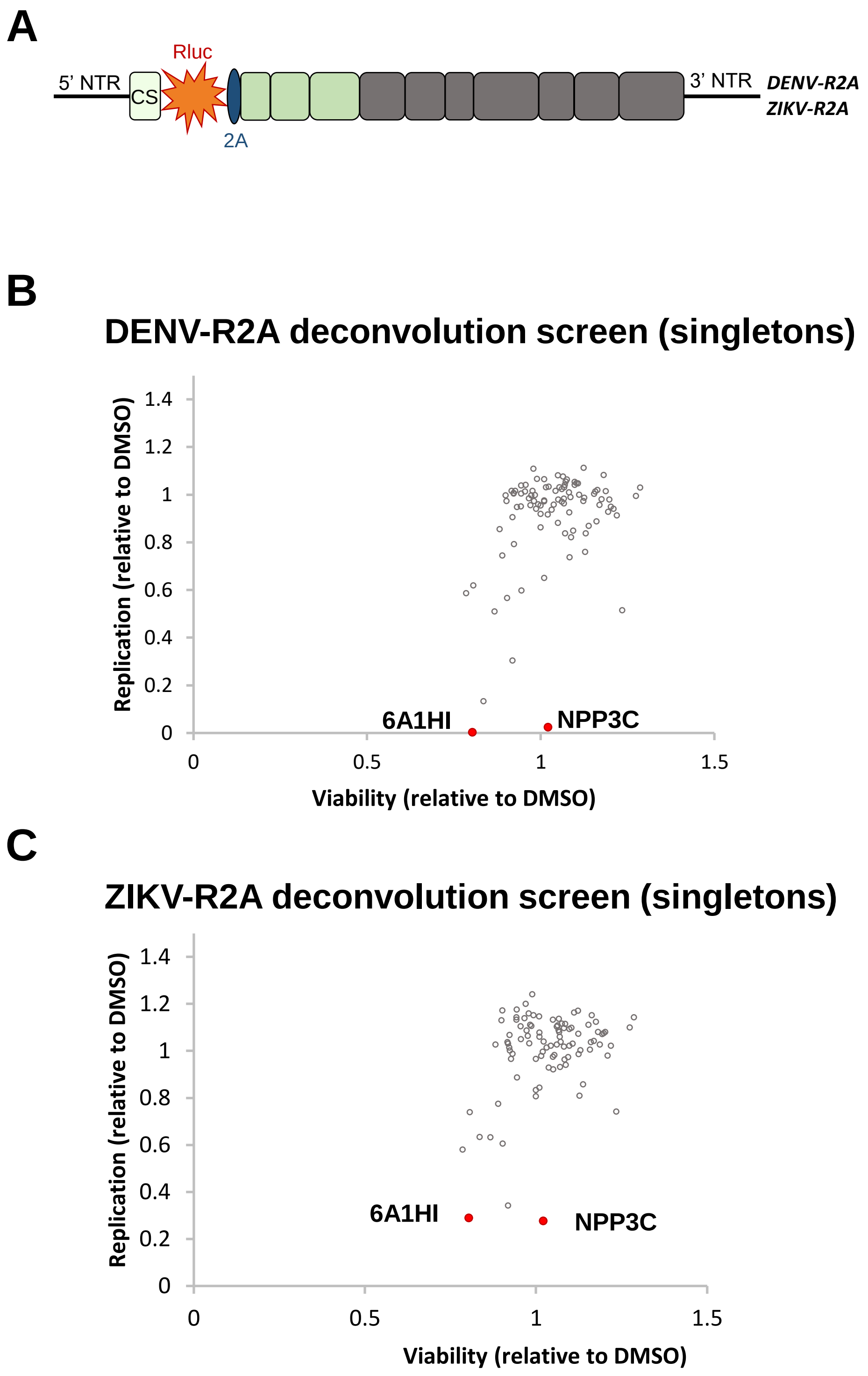

Supplemental Figure 1

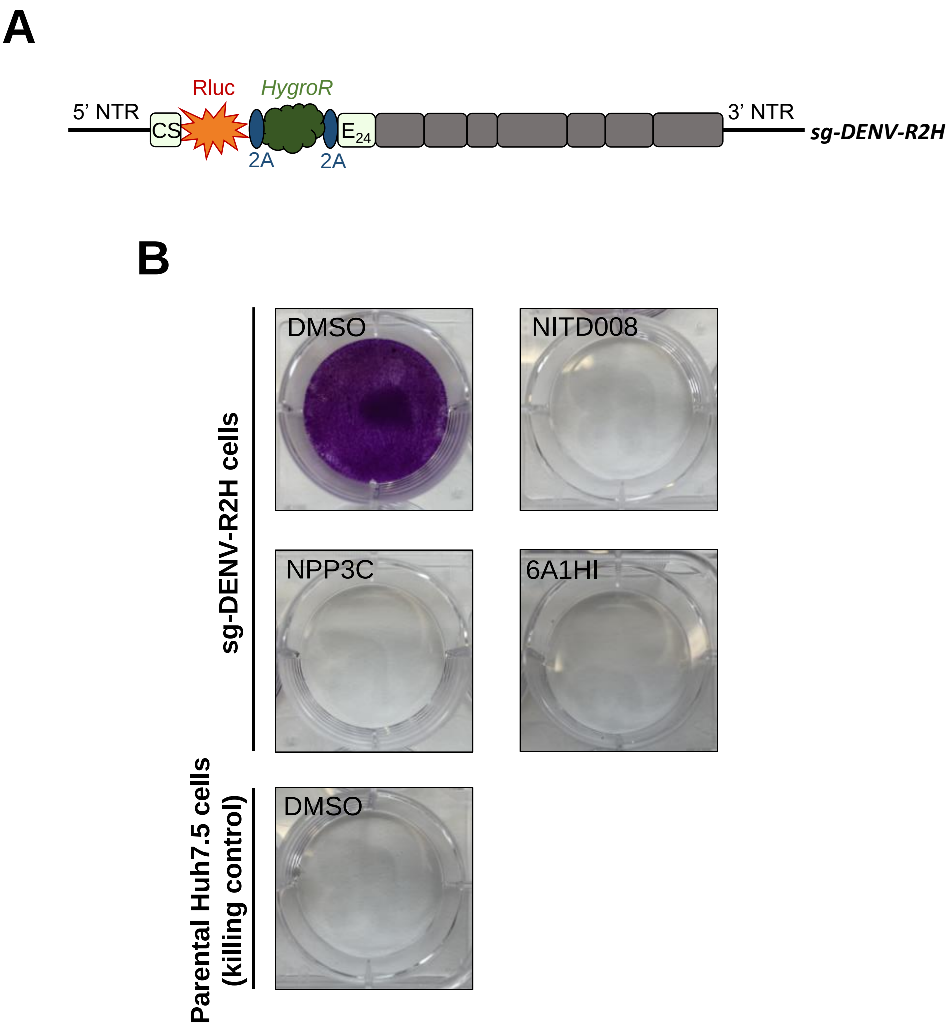

Supplemental Figure 2
